# MgADP Promotes Myosin Head Movement toward Actin at Low [Ca^2+^] to Increase Force Production and Ca^2+^-Sensitivity of Contraction in Permeabilized Porcine Myocardial Strips

**DOI:** 10.3390/ijms232315084

**Published:** 2022-12-01

**Authors:** Peter O. Awinda, Weikang Ma, Kyrah L. Turner, Jing Zhao, Henry Gong, Mindy S. Thompson, Kenneth S. Campbell, Thomas C. Irving, Bertrand C. W. Tanner

**Affiliations:** 1 Department of Integrative Physiology and Neuroscience, Washington State University, Pullman, WA 99164, USA; 2 The Biophysics Collaborative Access Team (BioCAT), Illinois Institute of Technology, Chicago, IL 60616, USA; 3 Department of Biology, Illinois Institute of Technology, Chicago, IL 60616, USA; 4 School of Molecular Biosciences, Washington State University, Pullman, WA 99164, USA; 5 College of Basic Medical Sciences, Dalian Medical University, Dalian 116044, China; 6 Division of Cardiovascular Medicine, University of Kentucky, Lexington, KY 40536, USA; 7 Department of Physiology, University of Kentucky, Lexington, KY 40536, USA

**Keywords:** cardiac muscle, X-ray diffraction, thick-filament structure, muscle mechanics, myofilament function

## Abstract

Myosin cross-bridges dissociate from actin following Mg^2+^-adenosine triphosphate (MgATP) binding. Myosin hydrolyses MgATP into inorganic phosphate (P_i_) and Mg^2+^-adenosine diphosphate (ADP), and release of these hydrolysis products drives chemo-mechanical energy transitions within the cross-bridge cycle to power muscle contraction. Some forms of heart disease are associated with metabolic or enzymatic dysregulation of the MgATP-MgADP nucleotide pool, resulting in elevated cytosolic [MgADP] and impaired muscle relaxation. We investigated the mechanical and structural effects of increasing [MgADP] in permeabilized myocardial strips from porcine left ventricle samples. Sarcomere length was set to 2.0 µm at 28 °C, and all solutions contained 3% dextran T-500 to compress myofilament lattice spacing to near-physiological values. Under relaxing low [Ca^2+^] conditions (pCa 8.0, where pCa = −log_10_[Ca^2+^]), tension increased as [MgADP] increased from 0-5 mM. Complementary small-angle X-ray diffraction measurements show that the equatorial intensity ratio, I_1,1_/I_1,0_, also increased as [MgADP] increased from 0 to 5 mM, indicating myosin head movement away from the thick-filament backbone towards the thin-filament. Ca^2+^-activated force-pCa measurements show that Ca^2+^-sensitivity of contraction increased with 5 mM MgADP, compared to 0 mM MgADP. These data show that MgADP augments tension at low [Ca^2+^] and Ca^2+^-sensitivity of contraction, suggesting that MgADP destabilizes the quasi-helically ordered myosin OFF state, thereby shifting the cross-bridge population towards the disordered myosin ON state. Together, these results indicate that MgADP enhances the probability of cross-bridge binding to actin due to enhancement of both thick and thin filament-based activation mechanisms.

## 1. Introduction

Cross-bridge binding and force production is driven by MgATP (ATP) hydrolysis and the release of its products MgADP (ADP) and inorganic phosphate (Pi). Normal cardiac muscle contraction relies upon multiple mechanisms to maintain appropriate levels of ATP, ADP, creatine phosphate (CP) and creatine phosphokinase (CPK) activity. Typically, cardiac [ATP] is high (7–10 mM) compared to [ADP] (of order 10 µM), largely due to CPK catalyzing the reaction between CP and ADP to regenerate ATP. Metabolic or enzymatic dysregulation associated with heart disease and aging result in lower [ATP], CP/ATP ratio, and total creatine level [1,2,3,4], with increased [ADP] slowing cross-bridge detachment and compromising muscle relaxation [5,6,7].

The effects of ADP on the myosin cross-bridge cycle and Ca^2+^-activated myocardial contraction are well established. In an isometric muscle fiber, ADP dissociation from strongly bound myosin cross-bridge heads is the rate limiting step of the cross-bridge cycle [8,9,10]. Increased [ADP], therefore, slows myosin detachment rate and prolongs the duration of myosin attachment and the force generated by strongly bound cross-bridges. The increased binding also increases the Ca^2+^-sensitivity of the force-pCa relationship [7,11,12,13,14,15,16] by augmenting cooperative activation of the thin filament [17,18,19,20].

X-ray diffraction studies suggest that activation of intact muscles, Ca^2+^-activation of permeabilized muscle fibers, and rigor activation all shift the relative proximity of myosin heads away from the thick-filament backbone, nearer to thin-filaments [21,22,23,24]. This structural response has traditionally been attributed to the increased strong cross-bridge binding associated with force generation. Recent biochemical studies using synthetic thick-filaments reconstituted from porcine myosin [25] showed that increasing [ADP] reduced the population of myosin heads in the energy sparing super-relaxed (SRX) state and increased the population of myosin heads in the disordered-relaxed state (DRX) [26,27]. The SRX state is thought to be equivalent to the quasi-helically ordered OFF-state of myosin, where heads lay close to the thick filament backbone. The DRX state is thought to be equivalent to the disordered, ON state of myosin, where myosin heads can bind with actin [28,29]. While these ADP-dependent increases in the ON-state myosin heads could feed into greater recruitment of actomyosin cross-bridges, the effects of increased [ADP] on thick-filament structure are not well-defined. To investigate the effects of ADP on thick-filament structure, and the production of force at low and activating Ca^2+^-concentrations, we performed a combination of small angle X-ray diffraction and muscle mechanics measurements in permeabilized myocardial strips from porcine cardiac ventricles.

## 2. Results

### 2.1. Effects of ADP on Myofilament Lattice Structure

**Figure 1:**
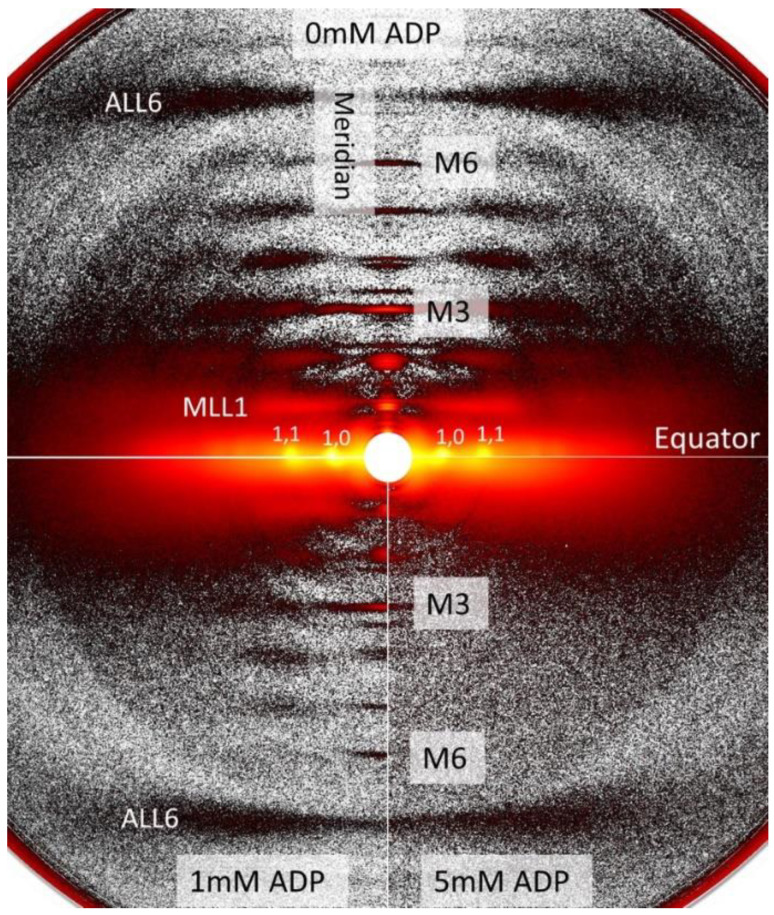
Representative X-ray diffraction patterns from permeabilized porcine myocardium at different concentrations of ADP. Characteristic relaxed patterns with intense layer line reflections in the absence of ADP (top panel). The myosin-based layer lines (e.g., MLL1) become weaker at 1 mM ADP (bottom left panel), and disappeared completely at 5 mM ADP (bottom right panel). The sixth order actin-based reflection (ALL6) remained relatively unchanged with changes in [ADP].

**Figure 2:**
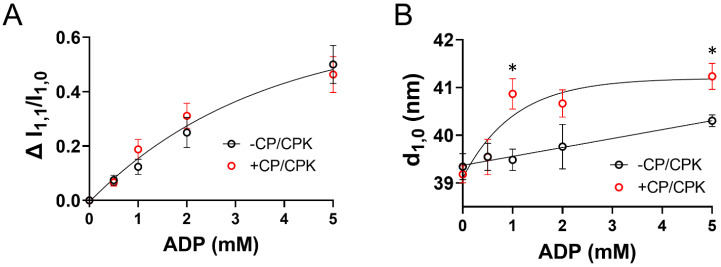
Equatorial reflection changes with increasing [ADP] under relaxing conditions (pCa 8.0). (**A**) Changes in the equatorial intensity ratio (ΔI_1,1_/I_1,0_) and (**B**) lattice spacing (d_1,0_) were plotted against [ADP], in the presence and absence of creatine phosphate (CP) and creatine phosphokinase (CPK). Two-way linear mixed models were utilized to characterize the main effects of ADP and CP/CPK and their interaction. Panel A depicts ΔI_1,1_/I_1,0_ relative to 0 mM [ADP] for each condition with a significant main effect for ADP (*p* < 0.001). In Panel B there were significant main effects of ADP (*p* < 0.001), CP/CPK (*p* = 0.004), and an ADP*CP/CPK interaction (*p* = 0.02), with * indicating a Fisher’s LSD post hoc difference between −CP/CPK and +CP/CPK at a single [ADP] at *p* < 0.05. 9 fibers were used for each different CP/CPK group.

We studied the X-ray diffraction patterns from relaxed permeabilized porcine myocardium at different [ADP]. Qualitatively, permeabilized porcine myocardium showed characteristic relaxed myosin based layer lines (MLL1 and MLL2) originating from the quasi-helically ordered arrangement of resting myosin heads [30] in the absence of ADP (Figure 1). The intensities of these reflections decreased as [ADP] increased, indicating a progressive loss of the helical ordering of the myosin heads. The equatorial intensity ratio (I_1,1_/I_1,0_), is an indicator of the proximity of myosin heads to actin [30], and changes in I_1,1_/I_1,0_ (ΔI_1,1_/I_1,0_) are a measure of radial movement of myosin heads towards actin from 0 mM [ADP] (Figure 2A). The relative increase in ΔI_1,1_/I_1,0_ was indistinguishable in the presence (+CP/CPK) and absence (−CP/CPK) of creatine phosphate (CP) and creatine phosphokinase (CPK) as [ADP] increased. There was a subtle increase in inter-filament lattice spacing with increasing [ADP] in the presence and absence of CP and CPK (Figure 2B). This increase in lattice spacing was linear with increasing [ADP] for the –CP/CPK group, and non-linear for the +CP/CPK group.

We were surprised by the similar responses for ∆I_1,1_/∆I_1,0_ between the measurements in the presence and absence of CP/CPK. With CP and CPK constituting the ATP regenerating system within some of our solutions, we assessed the alignment of [ADP] in our solution recipes and measured [ADP] using a colorimetric ADP assay (Table 1). In the absence of CP and CPK, data show good agreement between calculated [ADP] from the solution recipes and measured solution conditions for the relaxing and activating solutions. In the presence of CP and CPK there was little ADP in solution (~0.5 mM).

### 2.2. OFF to ON Transitions of Myosin Heads as a Function of [ADP]

**Figure 3:**
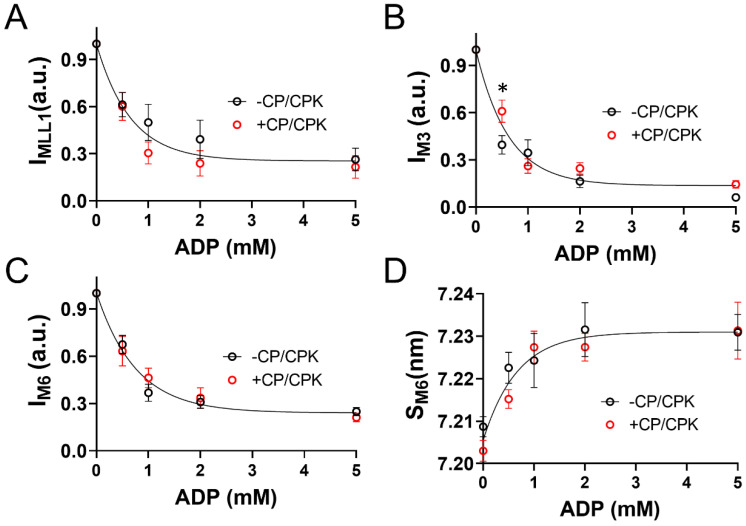
Myosin heads became more disordered as [ADP] increased under relaxing conditions. Intensity of the (**A**) first order myosin-based layer line (I_MLL1_), (**B**) third order myosin-based meridional reflections (I_M3_), (**C**) sixth order of myosin-based meridional reflection (I_M6_), and (**D**) spacing of the sixth order of myosin-based meridional reflection (S_M6_) were plotted against [ADP] in the presence and absence of CP and CPK. Intensity values were normalized to the 0 mM [ADP] value within each fiber for data shown in panels (**A**–**C**). Two-way linear mixed model analysis showed significant (*p* < 0.001) main effects of ADP for data in all panels. In Panel (**B**), there was also a main effect of CP/CPK (*p* = 0.03), with * indicating a Fisher’s LSD post hoc difference between −CP/CPK and +CP/CPK at a single [ADP] at *p* < 0.05. The number of fibers was 9 for each group.

A large portion of myosin heads in a relaxed muscle are quasi-helically ordered on the surface of the thick filament yielding the characteristic myosin-based layer line reflections in the in X-ray diffraction patterns [30]. Disordering of the myosin heads leads to an intensity decrease for the first order myosin-based layer line (I_MLL1_, Figure 3A) as [ADP] increased. The intensity of the M3 reflection (I_M3_) primarily stems from the myosin heads (1/14.3 nm^−1^), while the intensity of higher order M6 reflection arises from finer structures within the thick filament backbone (1/7.15 nm^−1^). Intensities of both M3 and M6 reflections also decreased as [ADP] increased (Figure 3B,C). Spacing of the M6 reflection also increased as [ADP] increased, indicating extension of the thick-filament backbone (Figure 3D). Surprisingly, there were no differences with or without CP/CPK for these myosin layer line reflections and M6 spacing, with a general fit to an exponential function representing each process as [ADP] increased.

### 2.3. Myocardial Mechanics at pCa 8.0

**Figure 4:**
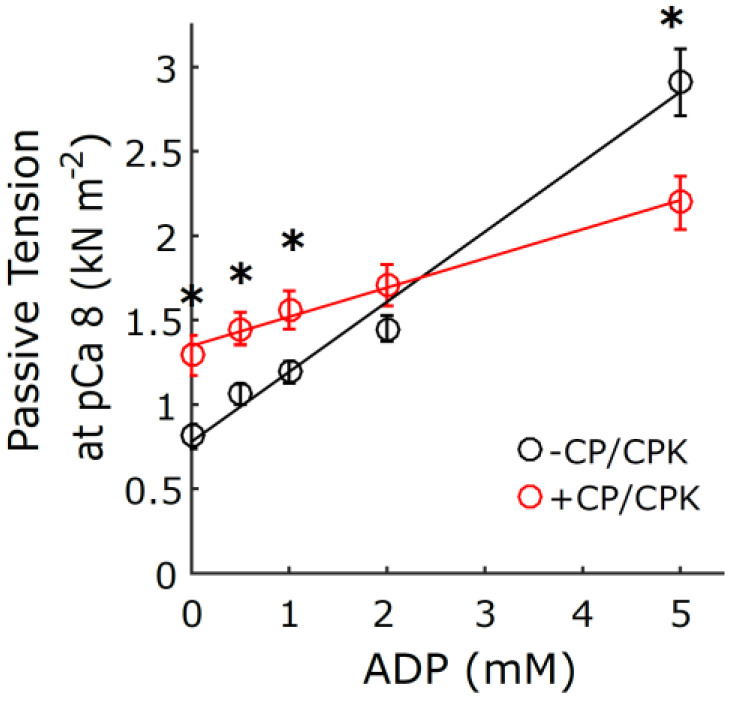
Tension increased with increased [ADP] under low [Ca^2+^] (pCa 8.0) relaxing conditions. Steady-state isometric stress (= force per cross-sectional area) was plotted against [ADP] in the presence and absence of CP and CPK. Nested two-way linear mixed models analysis showed main effects of ADP (*p* < 0.001), CP/CPK (*p* = 0.03), and an ADP*CP/CPK interaction (*p* < 0.001), with * indicating a Fisher’s LSD difference between −CP/CPK and +CP/CPK at a single [ADP] at *p* < 0.05. n fibers was 10 for each group, from two hearts.

**Figure 5:**
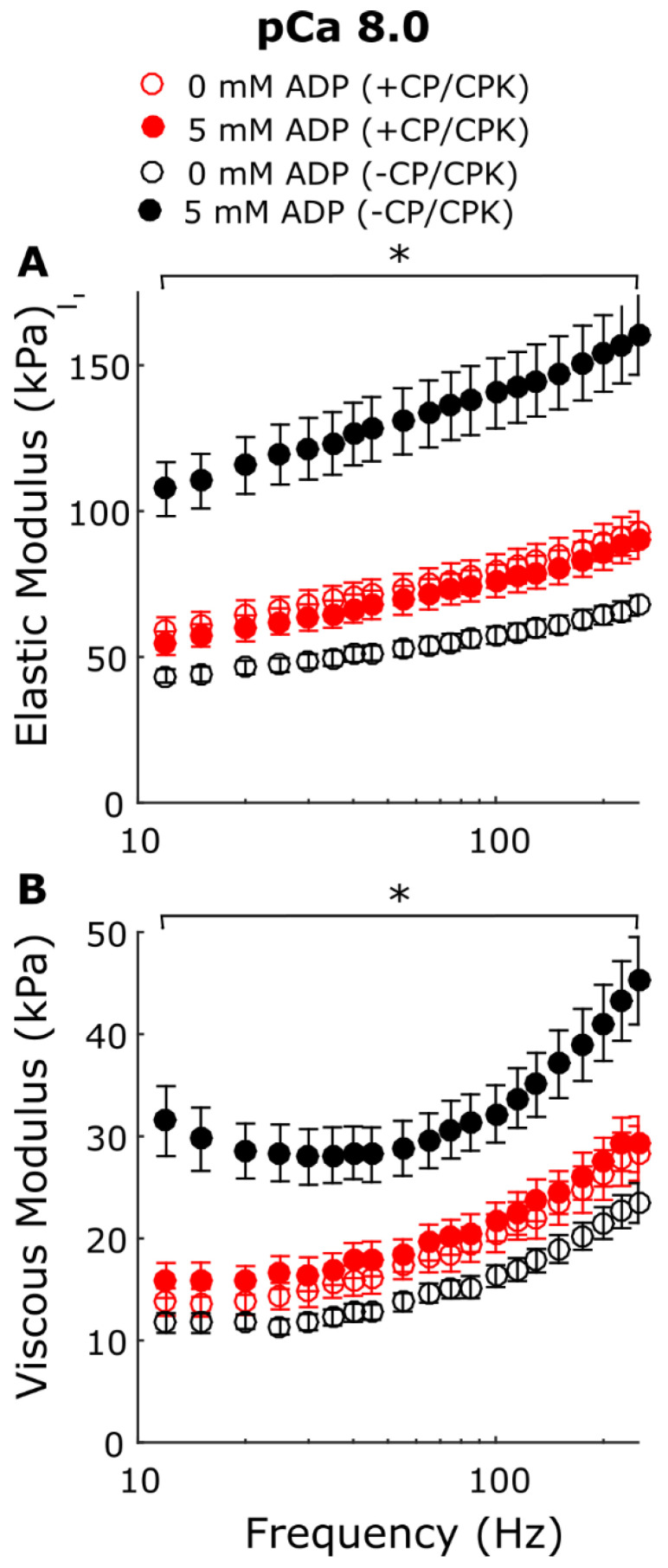
Viscoelastic myocardial stiffness under low [Ca^2+^] relaxing conditions. (**A**) Elastic and (**B**) viscous modulus values were plotted against frequency for 0 and 5 mM [ADP] in the presence and absence of CP and CPK. Nested two-way linear mixed models were utilized to characterize main effects frequency and treatment group ([ADP], with or without CP/CPK). There were significant main effects of frequency (*p* < 0.001) and treatment group (5 mM [ADP] without CP/CPK, *p* = 0.004) in Panel (**A**). A significant main effect of frequency (*p* < 0.001) and the frequency *CP/CPK interaction (*p* < 0.001) occurred for Panel (**B**). * indicates a Fisher’s LSD post hoc difference at a single frequency between the treatment group at 5 mM [ADP] without CP/CPK and all other treatment groups (*p* < 0.05). n fibers was 10 for each group, from 2 hearts.

In addition to the X-ray diffraction measurements under relaxing (pCa 8.0) conditions we performed mechanical measurements of permeabilized porcine myocardial strips at different [ADP]. Stress values increased linearly with increasing [ADP] in the presence and absence of CP and CPK at low [Ca^2+^] (Figure 4). The ADP-dependent increase in tension and range of tension values at low Ca^2+^ conditions was larger for the −CP/CPK strips than the +CP/CPK strips. Small-amplitude sinusoidal length-perturbations were used to measure the myocardial viscoelasticity under relaxing conditions (Figure 5). There were no significant differences in elastic and viscous moduli between 0 and 5 mM [ADP] in the presence of CP and CPK. In contrast, viscoelastic myocardial stiffness increased as [ADP] increased from 0 mM to 5 mM [ADP] in the absence of CP and CPK. The linearity of the elastic moduli vs. log_10_ frequency relationships are typical of relaxed myocardium, demonstrating little or no active cross-bridge cycling at pCa 8.0 (or much slower cycling where cross-bridges are bound for a prolonged duration, compared to normal, activated cycling) in the presence or absence of CP and CPK. For both groups, these stiffness data mirror the tension data, with the ADP-dependent increases in stiffness and range of stiffness values being larger for the −CP/CPK strips than the +CP/CPK strips (Figure 4).

### 2.4. Ca^2+^-Activated Myocardial Mechanics

**Figure 6:**
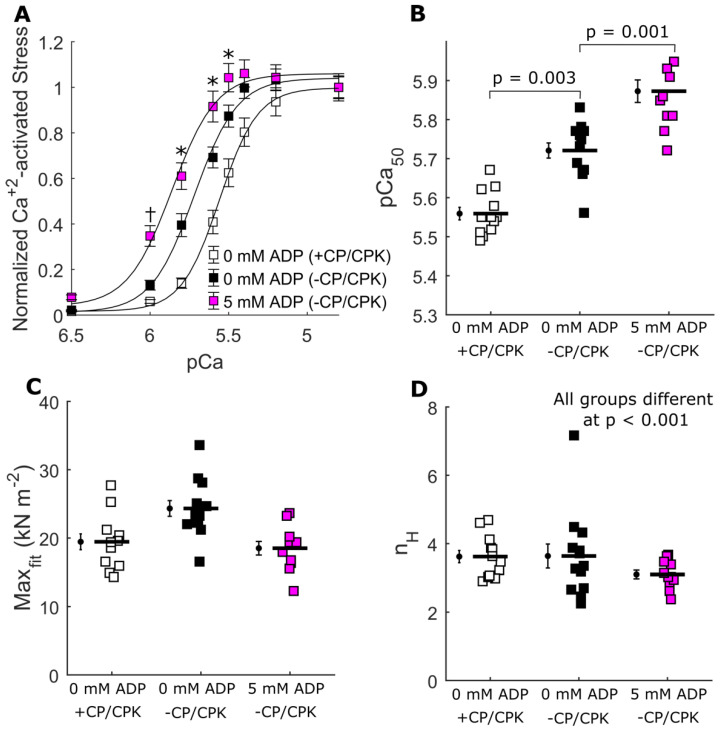
Ca^2+^-activated tension-pCa relationships. Steady-state isometric stress plotted against pCa, showing the increase in force from relaxing conditions at pCa 8.0 as strips were titrated to pCa 4.8. (**A**) Effects of [ADP] on Ca^2+^-activated force in the absence of CP and CPK, compared to control strips (=0 mM [ADP], +CP/CPK). Data were fit to a 3-parameter Hill equation, with parameters shown for (**B**) maximal Ca^2+^-activated stress (Max_fit_), (**C**) Hill coefficient (n_H_), and (**D**) calcium sensitivity(pCa_50_). Nested two-way linear mixed models analyses were used to characterize main effects of pCa and treatment group ([ADP] −/+ CPK). In Panel A, there was a significant main effect of pCa (*p* < 0.001), treatment group (*p* < 0.001), and a pCa* treatment group interaction (*p* < 0.001) were observed, with * indicating a Fisher’s LSD post hoc difference between all treatment groups at a single pCa (*p* < 0.05). † indicates a Fisher’s LSD post hoc difference between the treatment group of 5 mM ADP without CP/CPK and all other treatment groups at a single pCa (*p* < 0.05). Nested one-way linear mixed models were utilized to characterize the main effect of treatment group ([ADP] −/+ CPK) in Panels B, C, and D. This analysis showed a significant main effect of treatment group in panels B (*p* = 0.03), C (*p* = 0.03) and D (*p* < 0.001), with the *p*-values listed in each panel indicating Fisher’s LSD post hoc differences between groups. n fibers were 13 for 0 mM ADP (+CP/CPK), 12 for 0 mM ADP (+CP/CPK), and 11 for 5 mM ADP (+CP/CPK), from 2 hearts.

Figure 6 shows the effects of [ADP] on Ca^2+^-activated tension-pCa relationships in the presence and absence of CP and CPK. Compared to control strips (=0 mM ADP, +CP/CPK), both 0 and 5 mM ADP increased Ca^2+^-sensitivity of the tension-pCa relationships, as measured by the pCa_50_ values (Figure 6A,D). The effects of [ADP] on maximal Ca^2+^-activated stress were mixed for the −CP/CPK strips (Figure 6B), showing small differences among the three groups. Compared to control strips, maximal stress increased for the 0 mM ADP and decreased for the 5 mM ADP.

**Figure 7:**
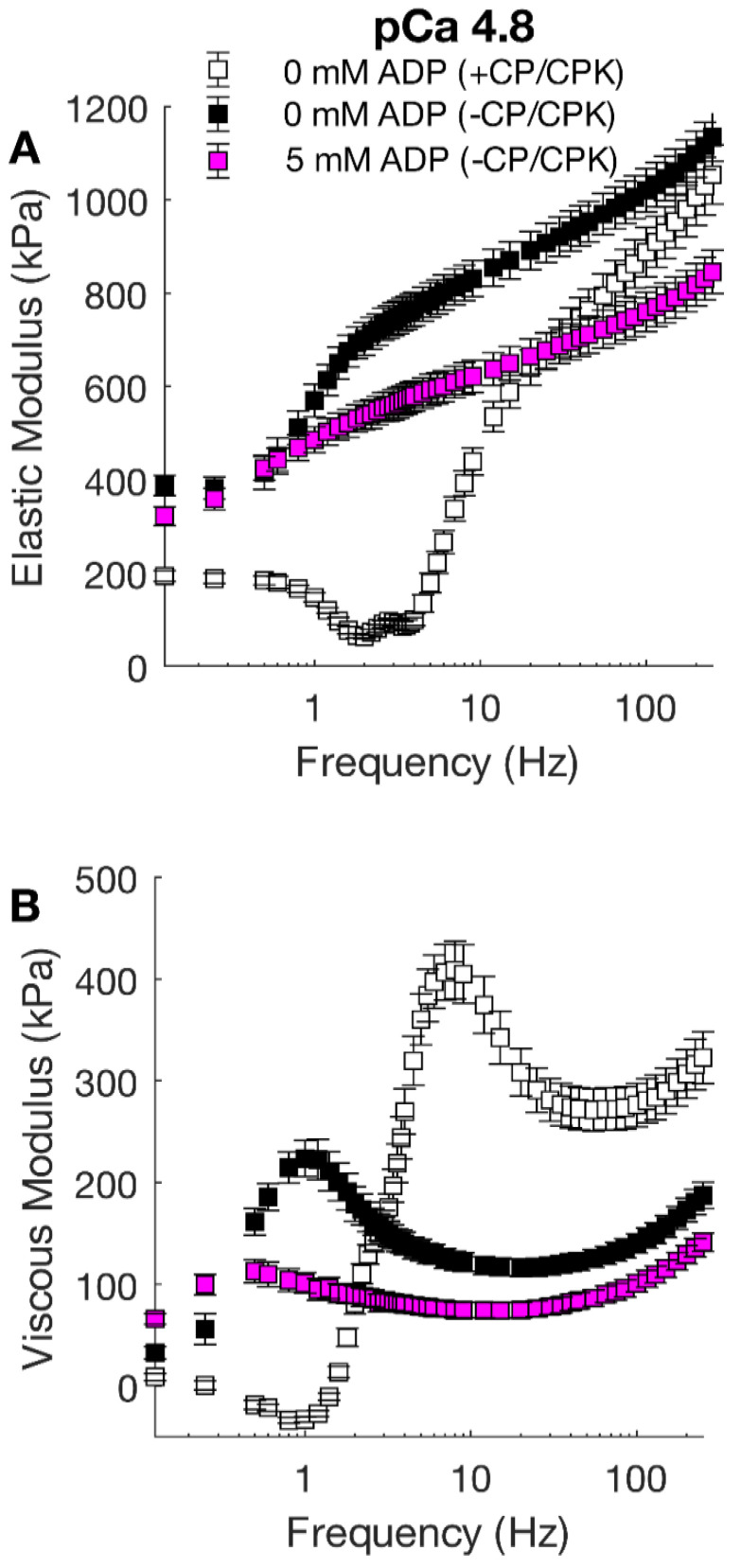
Viscoelastic myocardial stiffness at maximal Ca^2+^-activation. (**A**) Elastic and (**B**) viscous modulus values were plotted against frequency for 0 and 5 mM [ADP] in the presence and absence of CP and CPK at pCa 4.8. Nested two-way linear mixed models analysis was used to assess main effects of frequency, treatment group ([ADP] ± CP/CPK) and the frequency * treatment group interaction. In both panels there was a main effect of frequency (*p* < 0.001), treatment (*p* < 0.001), and their interaction (*p* < 0.001). Within both panels Fisher’s LSD post hoc analysis indicated all treatment groups were significantly different from each other at a single frequency (*p* < 0.05, where obviously different) except at intercept points where moduli values cross over each other. n fibers were 13 for 0 mM ADP (+CP/CPK), 12 for 0 mM ADP (+CP/CPK), and 11 for 5 mM ADP (+CP/CPK), from 2 hearts.

Small-amplitude sinusoidal length-perturbations were used to measure the viscoelasticity of these strips under Ca^2+^-activated conditions (Figure 7). Frequency-dependent shifts in the dips and peaks of the moduli-frequency relationships illustrate changes in the overall cross-bridge cycling rate. The fastest cross-bridge cycling occurred in control strips (0 mM ADP, +CP/CPK), with the moduli-frequency relationships being shifted towards higher frequencies. Compared to these control strips, removing CP/CPK slowed cross-bridge cycling kinetics, as evident from the shift toward lower frequencies for the moduli at 0 and 5 mM ADP. Consistent with this slowing of the cross-bridge cycling was increased cross-bridge binding, most easily shown by the increases in the magnitude of the elastic moduli for 0 and 5 mM ADP −CP/CPK strips. This slower cross-bridge cycling and increased cross-bridge binding act in unison to augment cross-bridge contributions to thin-filament activation, leading to the increased pCa_50_ values shown in Figure 6.

## 3. Discussion

### 3.1. Increased Tension and Stiffness with Increased [ADP]

The mechanics data presented here show that increasing [ADP] in permeabilized myocardial strips increases force and stiffness at low [Ca^2+^]. The greater forces induced by elevated [ADP] under relaxed conditions, sometimes called “ADP-contraction” [7,15], has been proposed to reflect increased levels of thin-filament activation by strongly bound myosin cross-bridges in the myosin-ADP state [17,18]. Measurements of viscoelastic myocardial stiffness can help to illustrate relative differences in the bound cross-bridge population under different conditions. Plotting the elastic and viscous moduli relationships against the log of frequency leads to a relatively ‘flat’ response as observed for all groups under relaxing conditions where there is little or no cross-bridge cycling (Figure 5). Consistent with larger increases in tension for the −CP/CPK fibers (Figure 4), the larger increases in moduli values between 0 and 5 mM ADP in the absence of CP and CPK indicates greater cross-bridge binding at 5 mM ADP. There were much smaller differences in viscoelastic myocardial stiffness for the other three groups (i.e., 0 mM ADP −CP/CPK and both +CP/CPK conditions), indicating cross-bridge binding of the same order of magnitude for these other three groups where solution [ADP] was low (<0.5 mM). Taken together, these tension and myocardial stiffness data are consistent with strong cross-bridge binding acting cooperatively to promote additional cross-bridge binding, even under relaxing conditions. This phenomenon appears to be suppressed in the +CP/CPK fibers compared to −CP/CPK fibers, but not completely eliminated. While ADP promotes formation of strong binding cross-bridges, elevated ATP in the presence of CP/CPK shifts myosin heads towards actin, as indicated by the increased I_1,1_/I_1,0_ at pCa 8. The close proximity of heads to actin may be expected to promote weak cross-bridge binding and could explain the residual stiffness in the +CP/CPK fibers, even under low [Ca^2+^] conditions. Increased numbers of weakly bound cross-bridges could lead to the formation of a few force-generating cross-bridges at high ADP concentrations. Even a small increase in force producing cross-bridges under “relaxed conditions” could increase passive force values, and could slightly increase stiffness as well.

### 3.2. Increased Calcium Sensitivity with Increased [ADP]

To assess the effect of increased [ADP] on calcium sensitivity we also measured force-pCa relationships, and viscoelastic myocardial stiffness at pCa 4.8, which also provides a metric of overall cross-bridge cycling rate. As typically found, the greatest effect of [ADP] was to augment the calcium-sensitivity (pCa_50_) estimated from the force-pCa relationship. A portion of this increased Ca^2+^-sensitivity can be explained by slower cross-bridge detachment in 0 and 5 mM [ADP] in the absence of CP/CPK, compared to control fibers (0 mM ADP in the presence of CP/CPK, Figure 6). As discussed above, slower cross-bridge detachment implies prolonged cross-bridge attachment which may be expected to amplify cooperative cross-bridge contributions to thin-filament activation and force production. These data are consistent with prior observations demonstrating that increases in [ADP] slow cross-bridge kinetics to increase cooperative cross-bridge contributions to activation of contraction [7,12,15,17,18]. 

The X-ray diffraction data presented here show that increasing [ADP] shifts a population of myosin heads from an ordered configuration near the thick-filament backbone to a less ordered configuration nearer to the thin-filament. It has been proposed that increased calcium sensitivity may not be solely a thin-filament-based phenomenon [31] and includes contributions from thick-filament-based activation processes. If so, our data showing an increase in thick-filament activation in the presence of elevated [ADP] (Figure 2 and Figure 3) could provide at least a partial explanation for the increased calcium sensitivity observed under these conditions. This interpretation is also supported by recent biochemical measurements showing that increased [ADP] reduced the population of myosin in the energy sparing super-relaxed state (SRX) relative to those in the disordered relaxed state (DRX), thought to be correlated with the ordered structure of the myosin OFF state and disordered structure of the myosin ON state, respectively [25]. Increased numbers of DRX myosin heads could promote greater cross-bridge binding and force production, consistent with our findings herein. Importantly, our measurements were performed at 28 °C, which may further imply that [ADP] affects thick-filament structure in vivo (37 °C), because myosin head organization decreases at lower temperature [32,33,34].

### 3.3. Increased Nucleotide Concentration Induces Structural Transitions in the Thick filaments

Our X-ray diffraction measurements of thick filament structural transitions were very similar whether CP and CPK were absent or present in the relaxing solutions. Albeit small in terms of absolute differences, thick-to-thin filament lattice spacing (d_1,0_) increased less as [ADP] increased when CP and CPK were absent (Figure 2B). This could be explained if a small number of bound myosin heads in the myosin-ADP state inhibits expansion of the lattice under relaxing conditions due to electrostatic repulsion between the myofilaments [35]. Smaller d_1,0_ values for the −CP/CPK fibers would be expected to increase the probability of cross-bridge binding [36], and consistently there were larger increases in tension as [ADP] increased without CP and CPK at low [Ca^2+^] (Figure 4). In the presence of CP and CPK it is likely that any added ADP in solutions would be converted to ATP by CP and CPK (Table 1) reducing the population of ADP-induced strongly bound cross-bridges. This would be expected to result in smaller increases in relaxed tension as [ADP] increased for +CP/CPK fibers. The reduced ADP-sensitivity to increases in tension could also follow, in part, from larger d_1,0_ values leading to less cross-bridge binding under relaxing conditions with CP and CPK (Figure 2B). The high degree of similarity between myosin thick-filament structural transitions in the presence or absence of CP/CPK implies that these transitions are sensitive to the total nucleotide pool independent of whether they comprise ADP or ATP. If so, this may provide a connection between the reduced ATP levels due to metabolic changes and depressed myocardium contractility [6,37].

## 4. Materials and Methods

### 4.1. Muscle Preparation for X-ray Diffraction

Frozen porcine wild-type left ventricular wall myocardium was provided by Exemplar Genetics LLC. Humane euthanasia and tissue collection procedures were approved by the Institutional Animal Care and Use Committees at Exemplar Genetics. Briefly, frozen porcine left ventricle wall was thawed in skinning solution (91 mM K^+^-propionate, 3.5 mM MgCl, 0.16 mM CaCl_2_, 7 mM EGTA, 2.5 mM Na_2_ATP, 15 mM creatine phosphate, 20 mM Imidazole, 30 mM BDM, 1% Triton-X100) at room temperature for 1 h before dissection into smaller strips. These myocardial strips were skinned overnight at 4 °C. The fiber bundles were further dissected into preparations with a length of 5 mm with a diameter of 200 µm prior to attachment of aluminum T-clips to both ends in pCa 8 solution with 3% dextran (91 mM K^+^-propionate, 3.5 mM MgCl_2_, 0.16 mM CaCl_2_, 7 mM EGTA, 2.5 mM Na_2_ATP, 15 mM creatine phosphate, 20 mM Imidazole, 30 mM BDM, 3% dextran) on ice for the day of the experiment. X-ray diffraction experiments were performed at the BioCAT beamline 18ID at the Advanced Photon Source, Argonne National Laboratory [38]. The X-ray beam energy was set to 12 keV (0.1033 nm wavelength) and the incident flux was set to ~5 × 10^12^ photons per second. The specimen to detector distance was about 3 m. The muscle was incubated in a customized chamber with one end attached to a force transducer (Model 402B Aurora Scientific Inc., Aurora, ON, Canada) and Kapton^TM^ windows in the X-ray path. The experiment was performed between 28 °C to 30 °C at a sarcomere length of 2.0 µm. X-ray diffraction patterns were collected on a MarCCD 165 detector (Rayonix Inc., Evanston, IL, USA) with a 1 s exposure time as a function of five increasing [ADP] (0, 0.5, 1, 2, and 5 mM) at pCa 8.0, with and without CP and CPK in the solutions. These solutions were shipped on dry ice from Washington State University to Argonne National Labs, and were identical to the solutions used for muscle mechanics experiments performed at Washington State University (described below). The muscle sample was oscillated along its horizontal axes at a velocity of 1 mm/s and moved vertically after each exposure to avoid overlapping X-ray exposures. Two to three patterns were collected under each condition and the X-ray reflection spacing and intensity data extracted from these patterns were averaged. 

### 4.2. X-ray Data Analysis

The data were analyzed using the MuscleX software package developed at BioCAT [39]. Briefly, the equatorial reflections were measured by the “Equator” routine in MuscleX as described previously [22]. The intensities and spacings of meridional and layer line reflections were measured by the “Projection Traces” routine in MuscleX as described previously [40,41]. 

### 4.3. Muscle Preparation for Mechanics

Frozen porcine myocardial samples were shipped from Argonne National Laboratory to Washington State University on dry ice and stored at −80 °C until they were thawed for mechanics experiments. In preparation for the mechanics experiments, frozen hearts were placed in a vial with dissecting solution and left on ice to thaw for 10 min. All solution concentrations are listed in mM unless otherwise noted. Dissecting solution: 50 N,N-bis(2-hydroxyethyl)-2-aminoethanesulfonic acid (=BES), 30.83 K propionate, 10 Na azide, 20 ethylene glycol-bis(2-aminoethylether)-N,N,N′,N′-tetraacetic acid (=EGTA), 6.29 MgCl_2_, 6.09 ATP, 1 1,4-dithiothreitol (=DTT), 20 2,3-butanedione monoxime(=BDM), 50 μM leupeptin, 275 μM pefabloc, and 1 μM transepoxysuccinyl-L-leucylamido(4-guanidino)butane (=E- 64). Once thawed, these samples were transferred to skinning solution (=dissecting solution with 1% TritonX *w*/*v* and 50% glycerol *w*/*v*) and pared down to myocardial strips roughly 180 µm in diameter and 700 µm in length, and skinned overnight at 4 °C. Skinned myocardial strips were then transferred to storage solution, and if not used immediately for mechanics experiments, strips were stored at −20 °C for 1–5 days until experiments were performed.

### 4.4. Muscle Mechanics

Aluminum t-clips were attached to both ends of skinned muscle strips and mounted between a piezoelectric motor (P841.40, Physik Instrumente, Auburn, MA, USA) and a strain gauge (AE801, Kronex, Walnut Creek, CA, USA) in relaxing solution. Normal relaxing solution: pCa 8.0, 20 BES, 5 EGTA, 5 MgATP, 1 Mg^2+^, 0.3 Pi, 35 CP, 300U/mL CPK, 200 ionic strength (adjusted with Na methane sulfonate), 3% dextran T-500 wt/vol, and pH 7.0. Sarcomere length was set to 2.0 µm and temperature was maintained at 28 °C throughout the entire experiment. Each strip underwent one of two types of experiments: (i) an ADP titration from 0 to 5 mM ADP, or (ii) a pCa titration from pCa 8.0 to 4.8. Other solutions used for mechanics experiments were: (i) normal relaxing solution with 5 mM ADP, (ii) relaxing solution at 0 and 5 mM ADP in the absence of CP and CPK, (iii) normal activating solution (=normal relaxing solution with pCa 4.8) at 0 and 5 mM ADP, and (iv) activating solution (=relaxing solution with pCa 4.8) at 0 and 5 mM ADP in the absence of CP and CPK.

Steady-state isometric tension (=force per cross-sectional area) was measured at each [ADP] or [Ca^2+^], and small amplitude sinusoidal length perturbations (amplitude = 0.125% muscle length) were used to measure myocardial viscoelasticity at pCa 8.0 or pCa 4.8. The stress–strain response was measured over a range of frequencies, and elastic and viscous moduli were calculated from the in-phase and out-of-phase portions of the stress–strain response.

Given that CP and CPK constitute the ATP regenerating systems within some of our solutions, we wanted to check similarities and differences between our solution recipes and measured [ADP] using a colorimetric ADP assay kit (Abcam, ab83359). Solutions were tested in duplicate (Table 1). In the absence of CP and CPK there was good agreement between the calculated [ADP] within the solution recipes and measured solution conditions for the relaxing and activating solutions. In the presence of CP and CPK, nominal levels of ADP (~0.5 mM) were measured for all the other solutions we tested.

### 4.5. Statistical Analysis

All data are listed or plotted as means ± SEM. Constrained nonlinear least squares fitting of the force-pCa data to a three-parameter Hill equation was performed using sequential quadratic programming methods in MATLAB (v.9.11.0, The MathWorks, Natick, MA). Statistical analyses were performed in SPSS using linear mixed models (IBM Statistical, Chicago, IL, USA). Linear mixed models incorporating two main effects (ADP and CP/CPK) and their interaction were used to analyze the equatorial and meridional parameters from X-ray diffraction data (because all fibers came from a single heart). Nested linear mixed models incorporating either one or two main effects were utilized for the muscle mechanics measurements (because fibers came from two hearts). Nested linear mixed model analysis links data from the same hearts, with each heart being a random effect, to optimize statistical power of the biological replicates (hearts) vs. the technical replicates (fibers). Post hoc analyses were performed using Fisher’s least significant difference (LSD) test; *p* values less than 0.05 were considered significant. The statistical test that we used and significant *p*-values, with post hocs, are listed in each figure legend.

## 5. Conclusions

In summary, we have complemented X-ray diffraction measurements of thick-filament structure with parallel muscle mechanics experiments to investigate the effects of increasing [ADP] in the presence and absence of CP/CPK. In the absence of CP and CPK, we find an increase in the level of thick-filament activation, evident by a decrease in the quasi-helical ordering of the myosin heads as [ADP] increases. This leads to increased stress under relaxed conditions, most likely due to increases in the numbers of strongly bound cross-bridges in the myosin-ADP state. Increased [ADP] also leads to increased Ca^2+^-sensitivity with contributions from both thick- and thin-filament based activation processes. In the presence of CP and CPK, there are comparable reductions of myosin head ordering as nucleotide concentration increased indicating that that the myosin OFF and ON structural transitions may be sensitive to the total nucleotide pool, and not to the chemical identity of the nucleotide. These findings could inform underlying mechanisms of contraction tied to metabolic or enzymatic dysregulation of [ATP], CP/ATP ratio, and total creatine levels associated with heart disease and aging.

## Figures and Tables

**Table 1:** Solution recipe conditions vs. measured [ADP].

pCa	CP (mM)	CPK (U/mL)	Solution Recipe ADP (mM)	Measured ADP (mM)
8	0	0	0	0.37 ± 0.00
8	0	0	5	5.16 ± 0.42
8	35	300	0	0.40 ± 0.03
8	35	300	2	0.45 ± 0.06
8	35	300	5	0.65 ± 0.13
4.8	0	0	0	0.48 ± 0.06
4.8	0	0	5	5.63 ± 1.49
4.8	35	300	0	0.51 ± 0.08

## Data Availability

All summarized data were presented in Figure 2, Figure 3, Figure 4, Figure 5, Figure 6 and Figure 7. Raw data are openly available in the supplemental data file associated with this manuscript (Supplementary Materials).

## Supplementary Materials

The following supporting information can be downloaded at: https://www.mdpi.com/article/10.3390/ijms232315084/s1.
